# Blockade of ICAM-1 Improves the Outcome of Polymicrobial Sepsis via Modulating Neutrophil Migration and Reversing Immunosuppression

**DOI:** 10.1155/2014/195290

**Published:** 2014-05-06

**Authors:** Yan-jun Zhao, Wen-jing Yi, Xiao-jian Wan, Jun Wang, Tian-zhu Tao, Jin-bao Li, Jia-feng Wang, Xiao-ming Deng

**Affiliations:** ^1^Department of Anesthesiology and Intensive Care, Changhai Hospital, Second Military Medical University, 168 Changhai Road, Shanghai 200433, China; ^2^Department of Anesthesiology and Intensive Care, Shanghai Ninth People's Hospital, Shanghai Jiaotong University, 639 Zhizaoju Road, Shanghai 200011, China

## Abstract

Intercellular adhesion molecule-1 (ICAM-1) is a key adhesion molecule mediating neutrophil migration and infiltration during sepsis. But its role in the outcome of sepsis remains contradictory. The current study was performed to investigate the role of anti-ICAM-1 antibody in the outcome of polymicrobial sepsis and sepsis-induced immune disturbance. Effect of anti-ICAM-1 antibody on outcome of sepsis induced by cecal ligation and puncture (CLP) was evaluated by the survival analysis, bacterial clearance, and lung injury. Its influence on neutrophil migration and infiltration, as well as lymphocyte status, in thymus and spleen was also investigated. The results demonstrated that ICAM-1 mRNA was upregulated in lung, thymus, and spleen of CLP mice. Anti-ICAM-1 antibody improved survival and bacterial clearance in CLP mice and attenuated lung injury. Migration of neutrophils to peritoneal cavity was enhanced while their infiltration into lung, thymus, and spleen was hampered by ICAM-1 blockade. Anti-ICAM-1 antibody also prevented sepsis-induced apoptosis in thymus and spleen. Positive costimulatory molecules including CD28, CD80, and CD86 were upregulated, while negative costimulatory molecules including PD-1 and PD-L1 were downregulated following anti-ICAM-1 antibody administration. In conclusion, ICAM-1 blockade may improve outcome of sepsis. The rationale may include the modulated neutrophil migration and the reversed immunosuppression.

## 1. Introduction

Sepsis refers to the systemic inflammatory response syndrome (SIRS) induced by infection. Severe sepsis, a more serious condition, is the combination of sepsis and dysfunction of at least one organ [1]. Despite the development of medical techniques, mortality of severe sepsis remains high which is over 40% according to the epidemiological studies from different countries [2–5]. Sepsis also costs a large amount of economic resources all over the world. New therapies are urgent for intervention of the progression of sepsis [2, 3].

Disturbance of the immune system is one of the most important features of sepsis, characterized by overwhelming inflammatory responses and dysfunction of the immune cells [6, 7]. Both anti-inflammatory agents and immune-enhancing treatment show ideal therapeutic effect in animals studies [8, 9], but none of these measures has been demonstrated to be effective in clinical trials [10]. The balance between anti- and proinflammatory responses becomes a key point in treating sepsis.

Intracellular adhesion molecule-1 (ICAM-1), also called CD54, is one of the mediators involved in leukocyte-endothelial interaction. After neutrophil rolling along the endothelium, CD18 complex on leukocyte may bind to ICAM-1 and promote adhesion and migration of leukocyte toward chemotactic agents [11]. It was reported that inhibition of ICAM-1 expression in lungs was associated with improvement of sepsis induced by cecal ligation and puncture (CLP) in mice, when they were treated by some agents such as protein kinase C-delta, hypertonic saline solution, and perfluorocarbon [12–14]. However, the direct role of ICAM-1 in polymicrobial sepsis remained controversial. Several studies used anti-ICAM-1 antibody or gene-deficiency animals to investigate the direct role of ICAM-1 in sepsis, but inconsistent results were found among them [15–18]. Some studies revealed that blockade of ICAM-1 decreased the survival rate in septic animals [15, 16], while others showed a beneficial role of ICAM-1 deficiency [17, 18]. van Griensven et al. [17] argued that the different model might be the reason of the contradictory results because some early studies use a model of bacterial injection, but they used a CLP model. However, Que et al. [15] identified that anti-ICAM-1 antibody or gene deficiency did not improve lung injury in the CLP model either.

Since ICAM-1 is a proadhesion molecule, its blockade using a specific antibody may hamper the proper migration of immune cells and development of lymphocyte. Thus, our present study was performed firstly to confirm the effect of ICAM-1 on polymicrobial sepsis and secondly to detect the apoptotic rate and expression levels of costimulatory molecules in thymus and spleen to clarify the effect of ICAM-1 on status of immune cells.

## 2. Materials and Methods

### 2.1. Mice and Cecal Ligation and Puncture Model

All animal experiments were approved by the Animal Care and Use Committee of Changhai Hospital. Male 8- to 10-week-old C57BL/6 mice (22–30 g) were purchased from the Animals Experimentation Center of Second Military Medical University. All mice were conditioned to the environment under controlled temperature (20 ± 2°C), humidity (60 ± 5%), and 12 h light/12 h dark cycle for one week before surgery.

CLP model was established as described previously [19]. In brief, mice were anesthetized with 2-3% sevoflurane and a midline abdominal incision was made after disinfecting the abdomen. After exposure, cecum was ligated with a 1-0 Prolene thread and punctured once with a 22-gauge needle. Then the cecum was replaced into the abdomen, and the peritoneal wall was closed in two layers. Sham-operated animals underwent similar laparotomy without ligation and puncture on the cecum. All animals were resuscitated by a subcutaneous injection of 1 mL sterile physiologic saline solution immediately after the surgery.

### 2.2. Drug Administration

Mice were randomly divided into four groups; (1) sham group: mice underwent the sham operation and received normal saline (200 *μ*L per mouse); (2) saline group: mice were subjected to CLP and received normal saline (200 *μ*L per mouse); (3) anti-ICAM-1 group: mice were subjected to CLP and intravenously injected with a functional grade purified anti-ICAM-1 antibody (eBioscience, USA) (50 *μ*g/200 *μ*L per mouse, about 2 mg/kg); (4) isotype group: mice were subjected to CLP and intravenously injected with rat IgG2a isotype antibody (eBioscience, USA) (50 *μ*g/200 *μ*L per mouse). Mice receive intravenously anti-ICAM-1 antibody, isotype antibody, or normal saline immediately after surgery.

### 2.3. Detection of mRNA by Reverse Transcription

Total RNA was extracted from the lung, thymus, and spleen using the TRIzol Reagent (Invitrogen) and the phenol-chloroform method 24 h after surgery. The cDNA was synthesized from total RNA in ABI PCR system using a PrimeScript 1st Strand cDNA Synthesis Kit (TaKaRa, China). The murine primer sequences are shown as follows:

 ICAM-1 (forward, F) 5′-ACAGACACTAGAGGAGTGAGCAGG-3′ and (reverse, R) 5′-GTGAGCGTCCATATTTAGGCATGG-3′;
 CD28 (F) 5′-CGGGAATGGGAATTTTACCT-3′ and (R) 5′-TTGACGTGCAGATTCCAGAG-3′;
 CD80 (F) 5′-CCATGTCCAAGGCTCATTCT-3′ and (R) 5′-TTCCCAGCAATGACAGACAG-3′;
 CD86 (F) 5′-TCAGTGATCGCCAACTTCAG-3′ and (R) 5′-TTAGGTTTCGGGTGACCTTG-3′;
 PD-1 (F) 5′-GGAGCAGAGCTCGTGGTAAC-3′ and (R) 5′-TACCAATGACCATGCCTTGA-3′;
 PD-L1 (F) 5′-TGCTGCATAATCAGCTACGG-3′ and (R) 5′-GCTGGTCACATTGAGAAGCA-3′;
 GAPDH (F) 5′-GGTCCTCAGTGTAGCCCAAG-3′ and (R) 5′-AATGTGTCCGTCGTGGATCT-3′.

The real-time PCR were conducted in the StepOnePlus Real-time PCR System (Applied Biosystems, USA) using a SYBR Premix Ex Taq II kit. PCR procedures were referred to the instruction provided by the manufacturer. The levels of mRNA were expressed as fold changes after normalization to GAPDH.

### 2.4. Survival Analysis and Bacterial Clearance

To evaluate the potential therapeutic effect of anti-ICAM-1 antibody against sepsis, 40 mice were randomly allocated to sham group, saline group, anti-ICAM-1 group, and isotype group; *n* = 10 for each group. Survival rates were assessed over the subsequent 7 days.

Blood and peritoneal lavage fluid (PLF) samples were collected 24 h after surgery from another 24 mice. Blood was collected by heart puncture after isoflurane anesthesia; *n* = 6 for each group. PLF was harvested after injection of 2 mL PBS into the peritoneum. Then 100 *μ*L aliquot of each dilution was spread on a tryptic soy agar (TSA) blood agar plate. All plates were incubated at 37°C for 24 h. Colonies were counted and the primary concentration of bacteria was quantified by multiplying with the dilution times. The bacterial burden was expressed as colony forming units (CFUs)/mL for all the samples.

### 2.5. Flow Cytometry

Neutrophil counts in PLF, thymus, and spleen 24 h after surgery were determined to investigate the migration of neutrophils; *n* = 6 for each group. The total cell number was counted after lysis of erythrocytes (for spleen and thymus, single-cell suspension was prepared). The neutrophil number was counted by staining with fluorochrome-conjugated anti-Gr-1 antibody. Cells were subjected to fluorescence-activated cell sorting and cell numbers were calculated by flow cytometry (MACSQuant, Miltenyi, German).

### 2.6. Histopathological Studies

The lungs of the mice in the four groups mentioned above were harvested 24 h after surgery for histopathological staining. These organ tissues were fixed in buffered formaldehyde (10% in PBS) for more than 8 h, dehydrated in graded ethanol, and embedded in paraffin. Four-micrometer sections were cut and paraffin was removed by xylene. The tissue sections were then stained with the hematoxylin and eosin reagent and observed under light microscopy. The criteria for scoring lung injury were as follows: 0, normal tissue; 1, minimal inflammatory change; 2, no obvious damage to the lung architecture; 3, thickening of the alveolar septae; 4, formation of nodules or areas of pneumonitis that distorted the normal architecture; 5, total obliteration of the field. The slides were examined by two pathologists who were unaware of the groups.

### 2.7. Measurement of the Wet-to-Dry Weight Ratio of Lung Tissues

Twenty-four hours after surgery, one lobe of the right lung was isolated, and the blood and water from the lung surface were removed to determine the wet weight. After the lobe was placed at 80°C for 48 hours, the dry weight was determined and the wet-to-dry ratio was calculated.

### 2.8. Myeloperoxidase (MPO) Activity Assay

The lungs were homogenized, centrifuged (40,000 rpm, 30 min, 4°C), and resuspended in 50 mM KPO_4_ buffer (PH 6.0) with 0.5% hexadecyltrimethylammonium bromide 24 h after surgery. Then, samples were sonicated and incubated at 60°C for 2 h. Later, samples were assayed for activity in a H_2_O_2_/O-dianisidine buffer at 460 nm with a spectrophotometer (Shanghai Precision & Scientific Instrument Co. Ltd, China). Results are expressed as units of MPO activity per gram of lung tissue.

### 2.9. Quantification of Apoptosis in the Spleen and Thymus

The thymus and spleen were harvested from septic and sham-operated mice and fixed with 10% buffered formalin 24 h after surgery. ApopTag Plus Peroxidase In Situ Apoptosis Detection Kit (Chemicon Billerica, MA, USA) was used for terminal deoxynucleotidyl transferase-mediated dUTP nick end labeling (TUNEL) staining. The sections of thymus and spleen were incubated in equilibration buffer for 10 minutes and then terminal deoxynucleotidyl transferase and dUTP-digoxigenin were added to the sections and incubated in a 37°C humidified chamber for 1 h. After stopping the reaction, the slices were washed and incubated with anti-digoxigenin-peroxidase solution, colorized with DAB/H_2_O_2_, and counterstained with bisbenzimide. Two investigators examined the samples in a blinded fashion. The percentage of the TUNEL-positive cells was used to represent the apoptosis rate.

### 2.10. Statistical Analysis

Data are expressed as the mean ± standard deviation (SD). All statistical analyses were performed in SPSS 16.0. Student's *t*-test was used for comparison between 2 groups and one-way analysis of variance was used for comparison between 3 or more groups. Kaplan-Meier method was utilized to compare the survival rate. A *P* level less than 0.05 was considered statistically significant.

## 3. Result

### 3.1. ICAM-1 mRNA Expression Is Upregulated in Lung, Thymus, and Spleen Tissue

**Figure 1:**
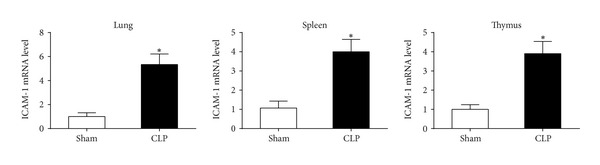
The expression levels of ICAM-1 mRNA were upregulated in lung, thymus, and spleen of CLP mice 24 h after CLP surgery. *n* = 6 per each group. _ _*P < 0.01 when septic mice were compared with sham-operated mice. Bars represent the mean ± SD.

CLP-induced polymicrobial sepsis resulted in upregulation of ICAM-1 in different organs 24 h after CLP surgery. As shown in Figure 1, mRNA levels of ICAM-1 increased more than 2-fold in lung, thymus, and spleen of CLP mice compared with those in sham-operated mice.

### 3.2. ICAM-1 Blockade Improved Survival and Bacterial Clearance in CLP Mice

**Figure 2:**
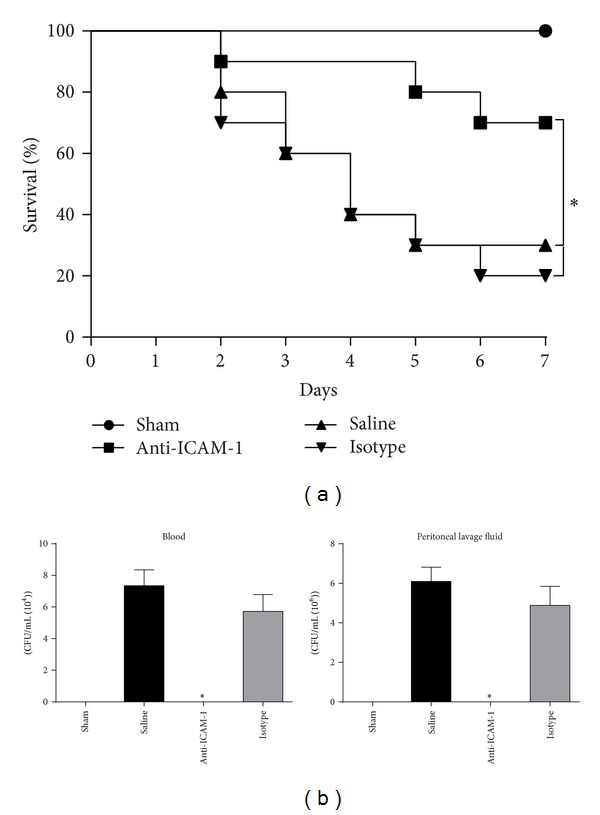
Anti-ICAM-1 antibody treatment improved survival (a) and bacterial clearance (b) in CLP mice. (a) The 7-day survival rates were compared among anti-ICAM-1 antibody treated CLP mice, isotype antibody treated CLP mice, saline treated mice, and sham-operated mice. *n* = 10 per each group. _ _*P < 0.05 when anti-ICAM-1 antibody treated mice were compared with mice treated with isotype antibody or saline. (b) Bacterial clearance was compared among anti-ICAM-1 antibody treated CLP mice, isotype antibody treated CLP mice, saline treated mice, and sham-operated mice 24 h after surgery. *n* = 6 per each group. PLF: peritoneal lavage fluid. _ _*P < 0.05 when anti-ICAM-1 antibody treated mice were compared with mice treated with isotype antibody or saline. Bars represent the mean ± SD.

The 7-day survival was assessed to investigate whether anti-ICAM-1 antibody protected mice from CLP-induced mortality. Survival of the mice in the anti-ICAM-1 group at the 7th day after surgery was 60%, significantly higher than in the CLP group (30%) and the isotype group (20%) (Figure 2(a)). Bacterial clearance was assessed in blood and PLF 24 h after surgery. Bacterial burden in blood and PLF was almost neglectable in the anti-ICAM-1 group, much lower than in the CLP group and the isotype group (Figure 2(b)).

### 3.3. Anti-ICAM-1 Antibody Treatment Attenuated Sepsis-Induced Lung Injury

**Figure 3:**
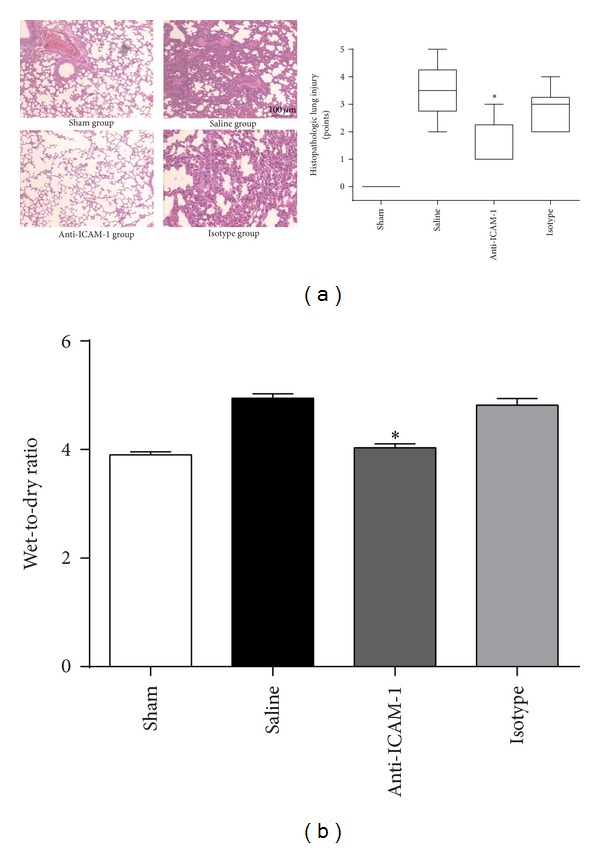
Anti-ICAM-1 antibody attenuated sepsis-induced lung injury as demonstrated by histopathological tests and wet-to-dry weight ratio. (a) The lungs of the anti-ICAM-1 antibody treated CLP mice, isotype antibody treated CLP mice, saline treated CLP mice, and sham-operated mice were harvested 24 h after surgery for histopathological staining. Histopathological tests showed milder impairment in lungs in anti-ICAM-1 group than in isotype and CLP groups. *n* = 6 per each group. (b) Lung wet-to-dry weight ratio was reduced in anti-ICAM-1 group than in isotype and saline groups. _ _*P < 0.05 when anti-ICAM-1 antibody treated mice were compared with mice treated with isotype antibody or saline. Bars represent the mean ± SD.

The histologic analysis of lung tissue showed that the lungs obtained from saline treated CLP mice were characterized by neutrophil infiltration, alveolar collapse, consolidation, and occasional alveolar hemorrhage. But anti-ICAM-1 antibody treatment significantly attenuated lung injury with intact pulmonary structure compared with isotype or saline treatment (Figure 3(a)). To further quantify the extent of lung edema, wet-to-dry weight ratio of lungs was measured 24 h after surgery. Reduced wet-to-dry weight ratio was observed in anti-ICAM-1 antibody treated mice than in isotype antibody and saline treated mice (Figure 3(b)).

### 3.4. Neutrophil Migration Was Modulated by Anti-ICAM-1 Antibody Treatment

**Figure 4:**
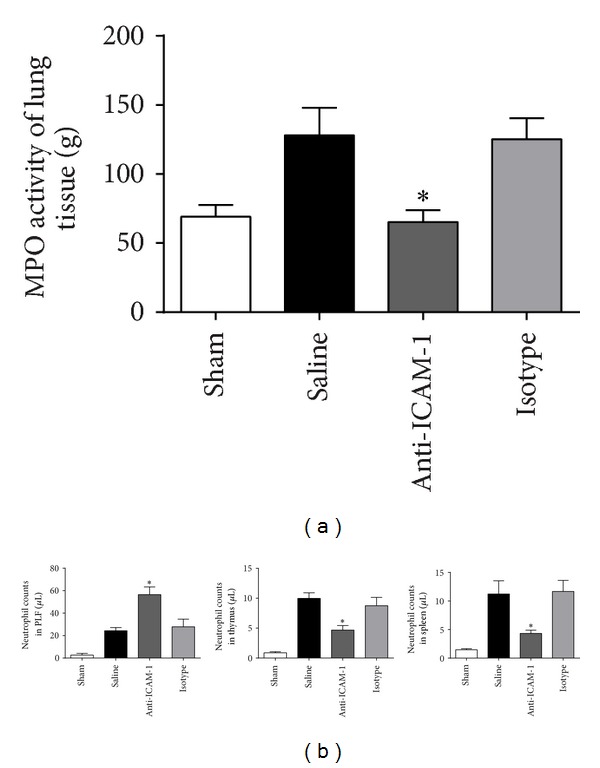
Anti-ICAM-1 blockade modulated neutrophils migration during sepsis. (a) MPO activity in lung homogenate was inhibited in the anti-ICAM-1 group; *n* = 6 per each group. _ _*P < 0.05 when anti-ICAM-1 antibody treated mice were compared with mice treated with isotype antibody or saline. Bars represent the median value and percentiles. (b) Neutrophil counts were elevated in PLF but reduced in thymus and spleen in the anti-ICAM-1 group compared with that in the isotype or saline groups, as demonstrated by flow cytometry. *n* = 6 per each group. _ _*P < 0.05 when anti-ICAM-1 antibody treated mice were compared with mice treated with isotype antibody or saline. Bars represent the mean ± SD.

Neutrophil migration was regarded as an important factor for killing bacteria, and it was also involved in pathogenesis of organ dysfunction induced by sepsis. Thus, we compared the MPO activity or neutrophil counts to evaluate the migratory ability of neutrophils in different loci 24 h after surgery. The assay of MPO activity in lung homogenate revealed that MPO activity in anti-ICAM-1 antibody treated mice was significantly inhibited compared with that in isotype antibody or saline treated mice (Figure 4(a)). Flow cytometry showed that neutrophil counts were elevated in PLF but reduced in thymus and spleen in anti-ICAM-1 group compared with that in the isotype and saline groups (Figure 4(b)).

### 3.5. Anti-ICAM-1 Antibody Improved the Immune Status in Thymus and Spleen

**Figure 5:**
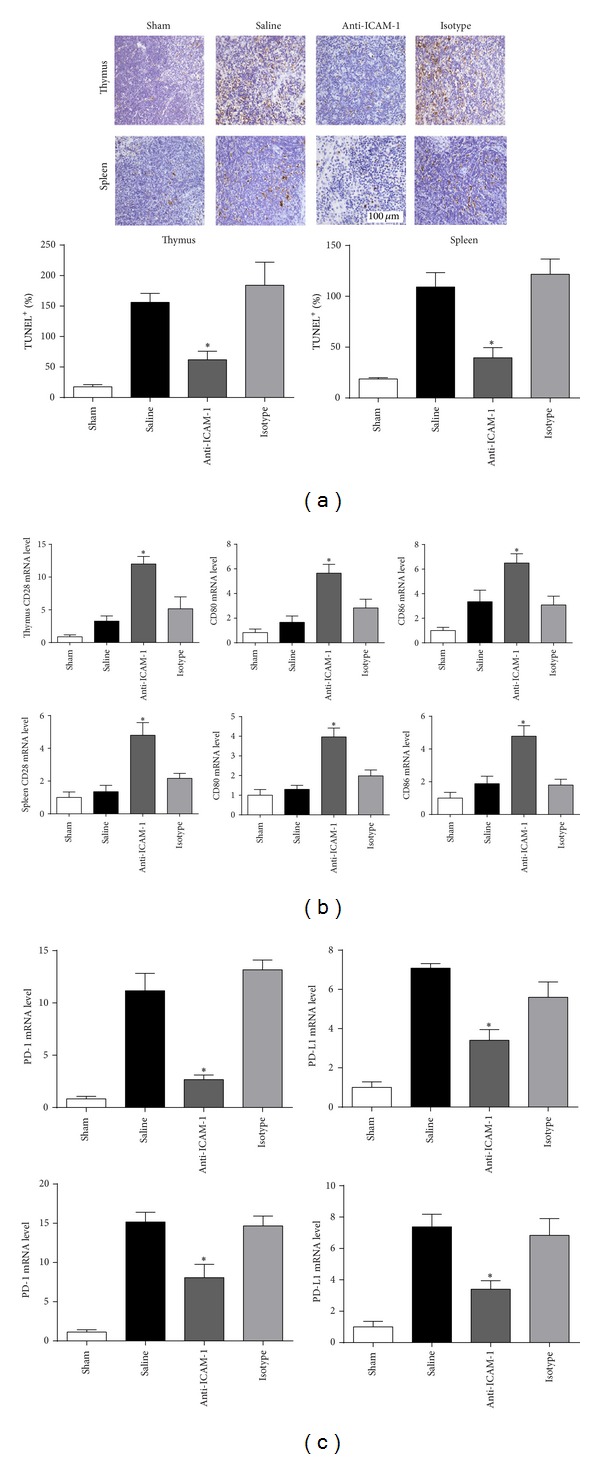
Effect of ICAM-1 blockade on the immune status in thymus and spleen. (a) Representative sections analyzed by the in situ TUNEL assays. Apoptotic cells were significantly reduced by anti-ICAM-1 treatment in thymus and spleen. (b) RT-PCR assays of costimulatory molecules in thymus and spleen, including CD28, CD80, CD86, PD-1, and PD-L1. *n* = 6 per each group. _ _*P < 0.05 when anti-ICAM-1 antibody treated mice were compared with mice treated with isotype antibody or saline. Bars represent the mean ± SD.

In order to investigate the effect of anti-ICAM-1 antibody on immune status, apoptosis and expression levels of costimulatory molecules in spleen and thymus were determined by TUNEL assay and RT-PCR, respectively. TUNEL assay showed that apoptotic cells were significantly less in anti-ICAM-1 group than in isotype and saline groups (Figure 5(a)) (*P* < 0.05). Positive costimulatory molecules, including CD28, CD80, and CD86, were all elevated (Figure 5(b)), while negative costimulatory molecules, including PD-1 and PD-L1, were reduced in thymus and spleen of anti-ICAM-1 antibody treated mice according to the RT-PCR assays (Figure 5(c)) (*P* < 0.05).

## 4. Discussion

Our present study reveals that ICAM-1 mRNA is upregulated in lung, thymus, and spleen during sepsis. Blockade of ICAM-1 using a specific antibody improves survival and bacterial clearance and attenuates sepsis-induced lung injury. Neutrophil infiltration into nonspecific target organs such as lung, thymus, and spleen is modified, while its migration to the peritoneal cavity is enhanced. Less infiltration of neutrophils into thymus and spleen may relieve sepsis-induced lymphocyte apoptosis and disturbance of costimulatory molecules, thereby modulating the immune dysfunction during sepsis.

Neutrophils are the first line of defense against bacterial infection. They respond to pathogens rapidly and produce reactive oxygen species to degrade bacteria [20]. Patients with neutropenia are believed to be more vulnerable to infection [21]. Unfortunately, activation and mobilization of neutrophils are always systemic during sepsis. At the same time of killing pathogens, neutrophils cause tissue damage as well. In the local site of infection, damage of tissue may be manifested as abscess. But in the remote nonspecific organs, organ dysfunction may develop, such as sepsis-induced lung injury, kidney injury, hepatic injury, and myocardial injury [22]. Removal of neutrophils from patients with systemic inflammation by filters has been shown to improve respiratory and renal function [23].

ICAM-1 is one of the most important adhesion molecules determining the adhesion and migration of neutrophils to target organs. However, overexpression of ICAM-1 on endothelial cells from nonspecific organs may be a leading cause of sepsis-induced organ dysfunction. Although we did not determine the protein level of ICAM-1, upregulation of ICAM-1 in organs had been well defined in many studies [24]. According to the study of Basit et al., anti-ICAM-1 reduced neutrophil recruitment in alveolar space after lipopolysaccharide (LPS) challenge by more than 50%. Antagonism of its ligand, CD11b, also reverses the endothelial injury caused by sepsis [25]. In our present study, anti-ICAM-1 antibody did attenuate sepsis-induced lung injury, as demonstrated by the histopathological test and wet-to-dry weight ratio.

However, it is very strange that early and late studies showed the absolutely converse effects of ICAM-1 on the outcome of sepsis. Two studies performed near the year of 2000 excluded any beneficial effect of anti-ICAM-1 antibody or ICAM-1 gene deficiency [15, 16]. There was even evidence showing a harmful effect on septic lung injury [15]. But two studies published in 2005 [18] and 2006 [17] demonstrated a protective role of ICAM-1 deficiency in polymicrobial sepsis. There might be some difference in techniques that we did not understand, including antibody craft and gene-deficiency technology. Anyway, there seemed to be more lines of evidence proving the protective role of ICAM-1 blockade in outcome of sepsis.

Modulation of neutrophil migration is a highlight in this study. Neutrophil paralysis is involved in the inability of bacterial clearance and organ dysfunction [26]. The modulatory effect of anti-ICAM-1 antibody enhances the migratory capacity of neutrophils to the infection locus while attenuating its infiltration to nonspecific organs. Chemotaxis of neutrophils is mediated by several factors, such as CXC chemokine receptors and C-C motif receptors. Unfortunately, most of the chemokine receptors are not specific and contribute to the impaired organs [27]. But our data showed that reduced neutrophil adhesion to endothelial cells by anti-ICAM-1 antibody increased the specificity of neutrophil migration. The chemokines for the chemokine receptors included several agents, such as ATP and cytokines. We speculated that inhibited neutrophil infiltration into nonspecific organs might reduce the concentration of chemokines in these organs but the concentration in infection locus remained unchanged. Therefore, migratory capacity of neutrophils towards infectious site was relatively enhanced by anti-ICAM-1 antibody. But we only evaluated the histological change of lung and liver, while dysfunction of other organs was also important during the pathogenesis of sepsis, such as heart, liver, and kidney. The role of anti-ICAM-1 antibody in sepsis-induced dysfunction of other organs remained to be further investigated.

It was also interesting to notice that lymphocyte apoptosis and disturbance of costimulatory molecules were reversed by anti-ICAM-1 antibody. Meanwhile, infiltration of neutrophils to the thymus and spleen was also attenuated, which might be the reason of the protective role of anti-ICAM-1 antibody in immune system. Immune dysfunction has been considered a main cause of death in septic patients and animals recently, manifested as lymphocyte apoptosis and disturbance of costimulatory molecules. Upregulation of coinhibitory molecules, PD-L1 and PD-1, and downregulation of positive costimulatory molecules, CD28, CD80, and CD86, are the features of sepsis-induced immunosuppression, reversal of which will improve the survival of septic mice [28, 29]. Recent studies tended to discover an inhibitory effect of neutrophils on lymphocyte. Pillay et al. [30] identified a new subset of neutrophils in human systemic inflammation, which inhibited lymphocyte proliferation stimulated by phytohemagglutinin. The marker of these neutrophils is CD11c^bright^/CD62L^dim⁡^/CD11b^bright^/CD16^bright^ and the inhibitory effect was mediated by the integrin Mac-1 (CD11b/CD18 complex). Neutrophils might also inhibit lymphocyte via other signals, as reported by the same group investigating the Mac-1, such as the coinhibitory signaling of PD-L1/PD-1 pathway. Interferon-*γ* upregulates the PD-L1 expression level on neutrophils, by which lymphocyte proliferation is inhibited [31]. Therefore, less infiltration of neutrophils into thymus and spleen might improve the immune environment of immune organs and reverse sepsis-induced lymphocyte dysfunction by attenuating neutrophil-lymphocyte interactions.

## 5. Conclusion

In conclusion, our present study demonstrates that elevation of ICAM-1 in lung, thymus, and spleen is detrimental in sepsis. Blockade of ICAM-1 using a neutralizing antibody attenuates sepsis-induced death and lung injury. Anti-ICAM-1 antibody modulates the migration and infiltration of neutrophils, which further contributes to less organ impairment and preserved lymphocyte function.
